# What drives researcher preferences for chemical compounds? Evidence from conjoint analysis

**DOI:** 10.1371/journal.pone.0294576

**Published:** 2023-11-27

**Authors:** Bongsuk Sung, Kang-Min Park, Chun Gun Park, Yong-Hee Kim, Jaeyong Lee, Tae-Eun Jin

**Affiliations:** 1 Department of International Trade, Kyonggi University, Suwon-si, Gyeonggi-do, Republic of Korea; 2 Korea Bioinformation Center (KOBIC), Korea Research Institute of Bioscience & Biotechnology (KRIBB), Daejeon, Republic of Korea; 3 Department of Mathematics, Kyonggi University, Suwon-si, Gyeonggi-do, Republic of Korea; 4 Department of Applied Statistics, Chung-Ang University, Seoul, Republic of Korea; Shimane Daigaku, JAPAN

## Abstract

We investigated the attributes and attribute levels that affect researcher preferences for chemical compounds. We conducted a conjoint analysis on survey data of Korean researchers using chemical compounds from the Korean Chemical Bank (KCB). The analysis estimated the part-worth utility for each attribute’s level, calculated relative importance of attributes, and classified user segmentation with different patterns. The results show that the structure database offers the highest part-worth utility to researchers, followed by high new functionality, price, screening service, and drug action data provided only by the KCB. Notably, researchers view the offer of a structured database and high new functionality as more important than other attributes in decision-making about research and development of chemical compounds. Furthermore, the results of segmentation analysis demonstrated that researchers have distinct usage patterns of chemical compounds: researchers consider structure database and high new functionality in cluster 1; and high new functionality and price in cluster 2, to be the most appealing. We discussed some policy and strategic implications based on the findings of this study and proposed some limitations.

## Introduction

A compound library is fundamental for developing new pharmaceutical drugs. Based on knowledge of the target protein and literature or patent precedents for chemical classes with possible activity at the drug target, various strategies of early-stage drug discovery, including high-throughput screening (HTS), fragment-based lead discovery (FBLD), encoded library technologies, and phenotypic approaches, have been implemented to identify new lead molecules and foster drug development [1]. The sheer number of molecules discovered necessitated the formation of extensive compound libraries representing the appropriate chemical space [2–4]. Therefore, public chemical banks have developed globally and competed to devise methods that can generate compound libraries with greater diversity [5]. Some examples include the US National Center for Advancing Translational Sciences (NCATS), European Lead Factory (ELF), Drug Discovery Initiative (DDI), and Korean Chemical Bank (KCB). Their efforts are aimed at promoting the use of chemical compounds, enhancing the efficiency, success, and commercialization of research and development (R&D) in the pharmaceutical industry. As such, chemical banks expend considerable effort encouraging researchers to utilize their chemical compounds. One of the best methods is supplying pharmaceutical researchers with compound libraries customized to their preferences [6–8].

Therefore, understanding what drives researcher preferences for chemical compounds is crucial. Economic theory posits that as a consumer, a researcher should express their preference based on the utility of chemical compounds, deciding whether to use or purchase by assigning value to specific attributes. Thus, economic valuation methods that assess preference based on perceived utility-based value can be applied to understand attributes affecting researcher inclinations for certain chemical compounds. These methods are also relevant because they focus on public or non-market goods such as those in provided by public chemical banks.

However, little attention has been paid to tackling the academic and practical significance of optimizing chemical compounds through economics-based approaches. This study attempts an economic estimation of attributes affecting researcher preferences for chemical compounds provided by a public chemical bank, KCB.

Economics is a utilitarian discipline that focuses on deriving the market value of goods based on their use by human society. The economic value of non-market goods, such as chemical compounds in the KCB, is the sum of use and non-use values [9–11]. However, using a chemical compound generally affects prices more strongly than non-use (i.e., the compound simply existing) [12]. Chemical research creates and enhances economic benefits via generating and utilizing compounds to solve problems in industrial sectors, such as drug development, food, security, health, climate change, and energy [13, 14]. Furthermore, as the sole public agency handling compound libraries in Korea, KCB is crucial to developing, collecting, and providing chemical compounds, enhancing drug R&D efficiency, and promoting commercialization [15, 16]. Thus, the value of chemical compounds is entirely associated with their utility for R&D and commercialization, not with their existence and preservation. Therefore, we employed a method for assessing the utility value of non-market goods to determine attributes affecting researcher preferences for chemical compounds.

A chemical compound consists of more than two attributes, including new functionality, drug action, and structural information; the part-worth of each attribute determines the compound’s total worth [17]. This calls for an analytic approach that can estimate importance and willingness-to-pay (WTP) per attribute. However, KCB does not give the true market price of chemical compounds when offering them to researchers, excluding maintenance, management, and labor costs. The price researchers see only accounts for the cost of dispensing tip, plate, tube cap, and sealing used for distribution of chemical compounds. Therefore, researchers know little about competitive market prices of chemical compounds, suggesting that it is a substantial burden for them to express WTP in monetary value. Although researchers provide WTP, their pricing may not consider all attributes and could be unreliable. In this situation, the best option is to take a stated preference approach [18] that allows researchers to select and rank the WTP of chemical compounds with different combinations of all attributes at various levels, with each level set to a fixed price based on expert opinion.

A conjoint analysis is ideal for empirically investigating the attribute and level that affect researcher preferences for chemical compounds in the KCB. Conjoint analysis assumes that the utility of a product or service can be divided into part-worth related to attribute levels [17, 19, 20]. Thus, conjoint analytical approaches can provide KCB managers with crucial information on the value researchers assign to specific attributes (importance weights) and their levels (part-worth utility) when assessing a chemical compound. Furthermore, conjoint analysis has the potential to address ambiguity, particularly necessary given that the pharmaceutical sector is substantially more likely than other industries to generate basic research that directly connects with successful new medication. In Korea, biotechnology firms tend to be small, providing economic rationale for government intervention and public R&D support. Beginning from 2021, when the Korea Drug Development Fund was set up, the government has been actively supportive of biotechnology companies and researchers in drug development. This process is divided into three stages: preclinical research, clinical-trials, and post-approval. Conjoint analysis can provide policymakers with data that informs the strategic focus of the publicly funded KCB. In the preclinical research phase, private companies and researchers who conduct drug discovery use various compound libraries to derive a candidate group of chemical compounds with the potential to become a new medicine. This process is expensive and time-intensive: preclinical research for drug development generally requires 31 months and 31% of total R&D costs on average [21]. Public chemical banks such as KCB can provide compound libraries needed by pharmaceutical researchers during R&D, contributing to lowering time and cost during preclinical research.

Using conjoint analysis, this study found that researchers who conduct R&D for drug development regard the structure database and high new functionality as the most important attributes of chemical compounds. Hence, compound libraries with these attributes are more likely to be employed for R&D during preclinical research. Providing appropriate compounds allows researchers to decrease time and costs associated with three steps: target identification/validation (determining the function of a causal agent in a given disease phenotype), hit identification (identifying compounds able to interact with the target), and lead optimization (improving multiple parameters of a preidentified lead compound) [22]. The freed resources can then be allocated to other challenges facing the pharmaceutical sector. Therefore, understanding researcher preferences for chemical compounds through a systematic conjoint analytical approach is a practical strategy for operating and managing public chemical banks that will encourage growth of the pharmaceutical industry.

In this study, we first described the theoretical basis of the conjoint preference model, identified the appropriate attributes of all included chemical compounds, and specified their attribute levels and dimensions. We then established an empirical model to examine the relationships between part-worth utilities of each attribute level and researcher preferences for chemical compounds. Second, we presented the survey framework, data collection, and sample characteristics. Third, we ran the model and presented the results. Fourth, we summarized the main findings of our analysis and their implications. Finally, we highlighted the study’s contributions and limitations.

## Conjoint preference model

The conjoint analytic approach estimates utility at an attribute level based on a part-worth function [20, 23]. We established the rating-based conjoint model and employed an ordinary least squares (OLS) regression to estimate part-worth utility, an additive value that sums to total utility of an attribute. The rating-based model is simple to apply [24, 25], employing interval and ratio scales to measure user preference correctly and precisely [17].

Before establishing a model, appropriate attributes and their levels should be specified to estimate an additive utility for an attribute. In this context, the attributes should reflect characteristics of chemical compounds and dimensions that are valuable to users. Among the methods for determining the most appropriate attribute, focus group discussions are particularly suitable in situations requiring specialized understanding, as is the case in this study [26, 27]. Focus groups are a mainstay for conjoint analysis because attributes and levels must be described in a way that is realistic and understood by the respondents [28–30].

In this study, we conducted a focus group discussion across six weeks, between July 12 and August 19, 2021. The focus group comprised seven researchers who used chemical compounds from KCB, including KCB researchers. The following rules were applied for identifying attributes and their levels: (a) each attribute must be independent from each other [31–33]; (b) the number of attributes cannot be more than seven [34]; (c) each attribute should be easily described [35]; (d) any attribute that affects utility should be included; (e) attributes must be meaningful and useful to respondents [28–30, 32]. The focus group identified the most suitable attributes and levels (per attribute) by sharing their experiences of using chemical compounds and analyzing public and private chemical libraries (NCATS, ELF, DDI, Enamine, Chemdiv, and KCB).

We performed a preliminary survey on the conjoint design during August 20–31, 2021. The preliminary survey showed that respondents did not have additional demands to improve the efficiency of conjoint questions. Furthermore, the preliminary test indicated that respondents never expressed a willingness to pay more for less desirable attributes. Such a reversal would have violated assumptions in standard economic theory and rational choice theory that regard the user or consumer of products and services as utility maximizers.

For each compound, we employed the attributes and levels extracted from the focus group as the basis for the conjoint study design. These attributes included new functionality (capacity to develop novel material), type based on acquisition path, availability of structure database, availability of drug action data, availability of screening service, and price (see Table 1).

**Table 1:** Attributes and levels of chemical compounds.

Attribute	Level		
	1	2	3
New functionality	Low	High	
Type	Deposited by researchers	Bought abroad	
Structure database	Not provided	Provided	
Drug action data	Not provided	Provided (only in literature)	Provided (only by KCB)
Screening service	Not provided	Provided	
Price	40,000 KRW	100,000 KRW	300,000 KRW

*Notes*: New functionality denotes the possibility of developing novel uses from the compound, such as new pesticides. Type denotes whether chemical compounds were purchased from overseas vendors or deposited by researchers conducting R&D. A structured database denotes whether researchers had access to a database of chemical structures. Drug action data refers to how the drug affects the body. A screening service denotes whether the drug had been screened by KCB. Prices denote the chemical price per 96-well plate (1 plate; 5 mM, 5 μL).

The following empirical model was established to examine relationships between part-worth utilities of each attribute level and researcher preferences for chemical compounds:

$$Y_{i}=\alpha_{0}+\sum_{j=1}^{2}\beta_{1j}X_{1j}+\sum_{j=1}^{2}\beta_{2j}X_{2j}+\sum_{j=1}^{2}\beta_{3j}X_{3j}+\sum_{j=1}^{3}\beta_{4j}X_{4j}+\sum_{j=1}^{2}\beta_{5j}X_{5j}+\sum_{j=1}^{3}\beta_{6j}X_{6j}+\epsilon_{i}$$

where *Y*_*i*_ is purchase probability rating given to each hypothetical compound alternative, *i*; *α*_0_ is the intercept; *β*_1*j*_ to *β*_5*j*_, and *β*_6_ are part-worth or utility value of the *j*th level of *i*th attribute; *X*_1*j*_ to *X*_5*j*_ are dummy variables of the *j*th level of *i*th attribute; *X*_6_ is price, which is a continuous variable. It estimates the effect of overall price as a linear coefficient. The consideration of a linear price parameter, as a common practice [36], reduces the number of parameters to be estimated and reduces the number ofdegrees of freedom in the estimation [37].

Considering the variables (Table 2), this study employed a re-expressed empirical model for estimation, as follows:

$$Y_{i}=\alpha_{0}+\beta_{12}X_{12}+\beta_{22}X_{22}+\beta_{32}X_{32}+\beta_{42}X_{42}+\beta_{43}X_{43}+\beta_{52}X_{52}+\beta_{6}X_{6}+\epsilon_{i}.$$

**Table 2:** Variables for the conjoint model.

Attribute	Level	Coding method		Variable
New functionality	Low	0		*X*_11_
	High	1		*X*_12_
Type	Deposited by researchers	0		*X*_21_
	Bought abroad	1		*X*_22_
Structure database	Not provided	0		*X*_31_
	Provided	1		*X*_32_
Drug action data	Not provided	0	0	*X*_41_
	Provided (only published in the literature)	1	0	*X*_42_
	Provided (not published in the literature but revealed only by KCB)	0	1	*X*_43_
Screening service	Not provided	0		*X*_51_
	Provided	1		*X*_52_
Price	40,000 KRW			*X*_6_
	100,000 KRW			
	300,000 KRW			

To provide KCB managers with crucial data on researcher chemical preferences as well as insights into their population segments, targeting, and positioning, we calculated the relative importance of each attribute, estimating the part-worth utility per attribute level. In addition, we defined the relative importance of attribute *I*_*i*_, taking into account the range of part-worths (*X*_*ij*_) across attribute levels, consistent with Raz et al. [38] and Sayadi et al. [17]: *I*_*i*_ = {*max*(*X*_*ij*_) − *min*(*X*_*ij*_)}, for each *i*. We ascertained the importance of *I*_*i*_ relative to other attributes via normalizing *I*_*i*_ based on the following equation: $W_{i}=\frac{I_{i}}{\sum_{i=1}^{n}I_{i}}$, where *n* is the number of attributes. Therefore, $\sum_{i=1}^{n}W_{i}=1$.

## Questionnaire framework, data collection and filtering, and sample characteristics

### Questionnaire framework

The conjoint analysis presents respondents with alternatives of chemical compounds designed through combining distinct levels of all attributes (not overlapping levels of the same attribute). The number of possible combinations in this study was 114 (= 2 × 2 × 2 × 3 × 2 × 3). Because respondents could not realistically be expected to answer all combinations, we used a fractional factorial design, widely acknowledged as the best way to test the effects of attributes on user preferences [39–41]. To construct the fractional factorial, we applied an orthogonal main-effect design that balanced the independent contributions of all main effects, assuming negligible interactions [42]. The analysis was performed in SPSS version 26. We generated 18 hypothetical profiles, including two holdout profiles serving as validation (Table 3).

**Table 3:** Chemical compound profiles.

Profile	NF	T	SD	DAD	SS	P
1	High	Deposited	Provided	Not provided	Not provided	100,000 KRW
2	Low	Deposited	Provided	Provided (L)	Not provided	40,000 KRW
3	High	Abroad	Provided	Provided (K)	Not provided	300,000 KRW
4	High	Deposited	Not provided	Not provided	Provided	40,000 KRW
5	High	Deposited	Provided	Provided (L)	Provided	300,000 KRW
6	Low	Deposited	Not provided	Provided (K)	Provided	100,000 KRW
7	Low	Deposited	Not provided	Not provided	Not provided	300,000 KRW
8	High	Deposited	Not provided	Provided (K)	Not provided	300,000 KRW
9	High	Abroad	Provided	Not provided	Provided	100,000 KRW
10	Low	Abroad	Provided	Provided (K)	Provided	40,000 KRW
11	Low	Deposited	Provided	Not provided	Provided	300,000 KRW
12	Low	Abroad	Not provided	Not provided	Provided	300,000 KRW
13	Low	Abroad	Not provided	Provided (L)	Not provided	100,000 KRW
14	High	Abroad	Not provided	Provided (L)	Provided	300,000 KRW
15	Low	Abroad	Provided	Not provided	Not provided	300,000 KRW
16	High	Abroad	Not provided	Not provided	Not provided	40,000 KRW
HO	High	Deposited	Provided	Not provided	Provided	300,000 KRW
HO	High	Abroad	Provided	Not provided	Not provided	300,000 KRW

*Notes*: Deposited, abroad, provided (L), and provided (K) denote deposition by researchers, bought abroad, provided (only published in the literature), and provided (only from KCB), respectively. NF, new functionality; T, type; SD, structural database; SS, screening service; DAD, drug action data; P, price per 96-well plate (1 plate; 5 mM, 5 μL); HO, holdout profile.

### Data collection and filtering

We collected data using a questionnaire-based survey containing questions about 18 hypothetical profiles. Researchers responded by entering purchase probability (ranging between zero and 10) for each profile. Data collection (including preliminary survey) complied with article 33 of the Statistics Act in Korea. Specifically, confidential information regarding individuals, corporations, and organizations that became known during in the course of producing statistics were protected and were not used for any purpose other than for this study. The authors’ institutions did not require ethical approval for statistical research, except in instances that deemed life-threatening or harmful to human subjects. Each participant provided informed consent regarding the study and protection of personal data.

To establish a sample framework for the survey, we first acquired a mailing list of 800 individuals who were using chemical compounds from the KCB. The survey was conducted online from September 1 to October 18, 2021. We created an online questionnaire and emailed it to the mailing list on September 1. We organized submitted questionnaires in the first round on September 15 and emailed the questionnaire to non-respondents. Return rate in the second round was very low. To enhance the return rate, the questionnaire was again sent to non-respondents using emails and mobile phone-based long text messaging service in the third (on September 27^th^) and fourth (on October 5^th^) rounds. We collected 151 responses for a response rate of 18.87%. After excluding 10 that were unsuitable for analysis, we aggregated 141 responses from the survey.

Next, we selected reliable observations from the survey responses to ensure reliability of conjoint analysis outcomes. We used three goodness-of-fit measures, consistent with prior studies [34, 43, 44]: Pearson and Kendall’s tau correlations for the estimation sample and Kendall’s tau correlations for the holdout sample, based on correlations between actual and predicted preference scores. To select candidate observations for deletion, we established a minimum Pearson correlation value using the adjusted *R*^2^ in multivariate regression, ${R^{2}}_{adjusted}=1-\left(\frac{\left(1-R^{2}\right)\left(N-1\right)}{N-P-1}\right)$, where *N* is the number of profiles per respondent, excluding holdout profiles, and *P* is the number of attributes. In the regression model with 16 profiles and six attributes, the adjusted *R*^2^ is approximately 0.399, which sets a minimum correlation of 0.632 (the square root of 0.399, so that the adjusted *R*^2^ would always be greater than zero). For the holdout sample, the minimum threshold of Kendall’s tau correlation was set at 0.400. These assessments yielded 130 usable responses for the conjoint analysis.

### Sample characteristics

Male and female researchers accounted for 70% and 30% of the sample, respectively (Table 4). Thirty researchers (23%) were below 40 years old. Approximately 77% were over 40 years of age: 44, 42, and 14 researchers were 40–49, 50–59, and > 60 years, respectively. In addition, 62 researchers (47.7%) were from research institutes, 42 (32.3%) were from universities, and 22 (20.0%) worked for private firms. Approximately 80% (103 respondents) had used chemical compounds only for R&D. Three respondents used chemical compounds only for commercial purposes, whereas 24 respondents used them for both R&D and commercial purposes.

**Table 4:** Descriptive statistics.

Division		Frequency	Ratio (%)	Total
Gender	Male	91	70.0	130
	Female	39	30.0	
Age	20–29	1	0.8	130
	30–39	29	22.3	
	40–49	44	33.8	
	50–59	42	32.3	
	Over 60	14	10.8	
Institution type	University	42	32.3	130
	Firm	26	20.0	
	Research Institute	62	47.7	
Main use of chemical compounds	For R&D	103	79.2	130
	For commercial	3	2.3	
	For R&D and commercial	24	18.5	

## Empirical analysis and results

The results of our part-worth utility estimate for full sample using OLS showed that only the regression coefficient of variable *X*_22_ was not significant (Table 5). Hence, excluding chemical compound type, the levels of other attributes provided positive part-worth utility to researchers. Therefore, increases in new functionality, structure database, drug action data, and screening service should positively affect marginal willingness to pay for chemical compounds.

**Table 5:** Part-worth utility function.

Independent Variables	Dependent Variable = *Y*_*i*_		
	Coefficient	Standard error	P-value
*Constant*	3.102	0.214	0.000
*X*_12_ (new functionality: high)	1.972	0.117	0.000
*X*_22_ (type: bought abroad)	0.155	0.117	0.118
*X*_32_ (structure database: provided)	1.984	0.117	0.000
*X*_42_ (drug action data (provided): from literature only)	0.408	0.144	0.003
*X*_43_ (drug action data (provided): from KCB only)	0.521	0.144	0.000
*X*_52_ (screening service: provided)	0.457	0.117	0.000
*X*_6_ (price)	-0.216	0.071	0.002

*Notes*: The function represents the full sample. Part-worth utility functions for the two clusters are available from authors upon request.

Estimation of part-worth utility for attribute level and relative importance for full sample revealed that researchers have the highest preference for chemical compounds with KCB-provided structure databases, followed by high new functionality, offer of screening services, drug action data from KCB, and drug action data from the literature. After normalizing relative importance of each attribute, we found that researchers considered structure database (35.8%) and new functionality (36.0%) as the most important attributes when utilizing chemical compounds, followed by drug action data (13.2%), screening service (8.3%), and price (9.2%) (part-worth utility for full sample are available from authors upon request).

We performed cluster analysis to understand how part-worth utility of attribute levels and their relative importance vary. We conducted hierarchical clustering with Euclidian distance between observations and the Ward criterion. We followed previous recommendations [45] for clustering analysis on a relatively small sample size and novel data, as this study was the first to attempt utility-based user segmentation of chemical compounds. The cluster analysis divided researchers (n = 130) into two categories based on part-worth utilities for attribute levels at the individual level (Table 6; for the distribution characteristics of users by cluster, see S1 Table). The part-worth utility is presumed to be heterogeneous in the individuals’ preference for the levels of each attribute [46, 47]. This implies that it is crucial to estimate the heterogeneity of part-worths at the individual level in the conjoint segment procedure. In the traditional rating-based segmentation procedure, an individual-level part-worth utility to group respondents has been estimated using OLS regression [48]. When a requisite number of response data on profiles and observations are obtained in correspondence with the number of parameters, OLS can be the most straightforward way of estimating part-worth utilities at the individual level [49]. Despite the simplicity of application, OLS regression generates unreliable individual-level part-worth utilities due to response biases due to more profiles than attributes [50]. Therefore, performing segmentation based on OLS-based individual-level part-worths may cause misclassification of respondents [51]. The challenge of OLS regression in estimating part-worth utilities at the individual level can be addressed using the part-worths of all the respondents in the sample and employing Hierarchical Bayes (HB) methods for estimation of the part-worths [47, 49]. HB techniques can facilitate obtaining of more accurate individual-level part-worth estimates in both rating- and choice-based conjoint models than in the OLS method [52]. To represent the heterogeneity in the preferences of individuals for the attributes, the traditional approach, OLS regression, applies a single normal distribution. In that case, the individual-level estimates tend to converge toward the population mean, which may veil potential heterogeneity in the individual preferences [53]. However, the HB technique can relax this weakness by considering heterogeneous preference across individuals [46, 47, 49, 53]. HB methods derive individual part-worths by combining information on the normal distribution across respondents. The distributions of the parameter are specified, which are updated using the Bayes theorem. HB methods employ computationally intensive techniques, including Markov Chain Monte Carlo simulation techniques, such as Gibbs sampling and Metropolis-Hasting algorithms, to generate estimates of the part-worths and standard errors of each respondent [49]. Consequently, the HB technique was employed to obtain individual-level part-worth estimates and estimate-based cluster analysis was performed to group the respondents with similar part-worths or importance values to identify segments. The results of the cluster analysis are presented in Table 6.

**Table 6:** Part-worth utility estimates for each level for all attributes and importance values of attributes by cluster.

Attribute	Attribute level	Cluster 1		Cluster 2	
		A	B (%)	A	B (%)
New functionality	Low	-2.528	33.5	-1.190	41.0
	High	2.528		1.190	
Type	Deposited by researchers	-0.433	5.7	0.236	0*
	Bought abroad	0.433		-0.236	
Structure database	Not provided	-3.100	41.2	-0.412	14.2
	Provided	3.100		0.412	
Drug action data	Not provided	-1.194	12.3	-0.556	15.2
	Provided (from literature only)	0.536		0.227	
	Provided (from KCB only)	0.658		0.329	
Screening service	Not provided	-0.554	7.3	-0.319	11.0
	Provided	0.554		0.319	
Price	40,000 KRW	0.013	0*	-0.539	18.6
	100,000 KRW	0.027		-1.077	
	300,000 KRW	0.040		-1.616	
Intercept		0.820		6.313	
Number of cases		76 (58.5%)		54 (41.5%)	

*Notes*: A and B denote part-worth utility estimates and importance values, respectively.

*presents statistically insignificant results from the estimation of part-worth utility function of Cluster 1 and Cluster 2, respectively.

Cluster 1 included 76 researchers (58.5% of entire sample) who considered the structure database (41.2%) highly appealing. The second-most preferred attribute was high new functionality (33.5%), followed by drug action data (12.3%), screening service (7.3%), and bought abroad (5.7%). Cluster 2 included 54 researchers (41.5% of entire sample); the most appealing attribute was high new functionality (41.0%), followed by price (18.6%), drug action data (15.2%), structure database (14.2%), and screening service (11.2%). Table 6 shows that price in cluster 1 and type of chemical compound (whether chemical compounds are deposited by researchers or bought abroad) in cluster 2 are statistically insignificant in the estimation of part-worth function, which indicate that price and type of chemical compound are not appealing attributes to the researchers in cluster 1 and cluster 2, respectively, and do not determine their preference when purchasing chemical compounds.

**Fig 1:**
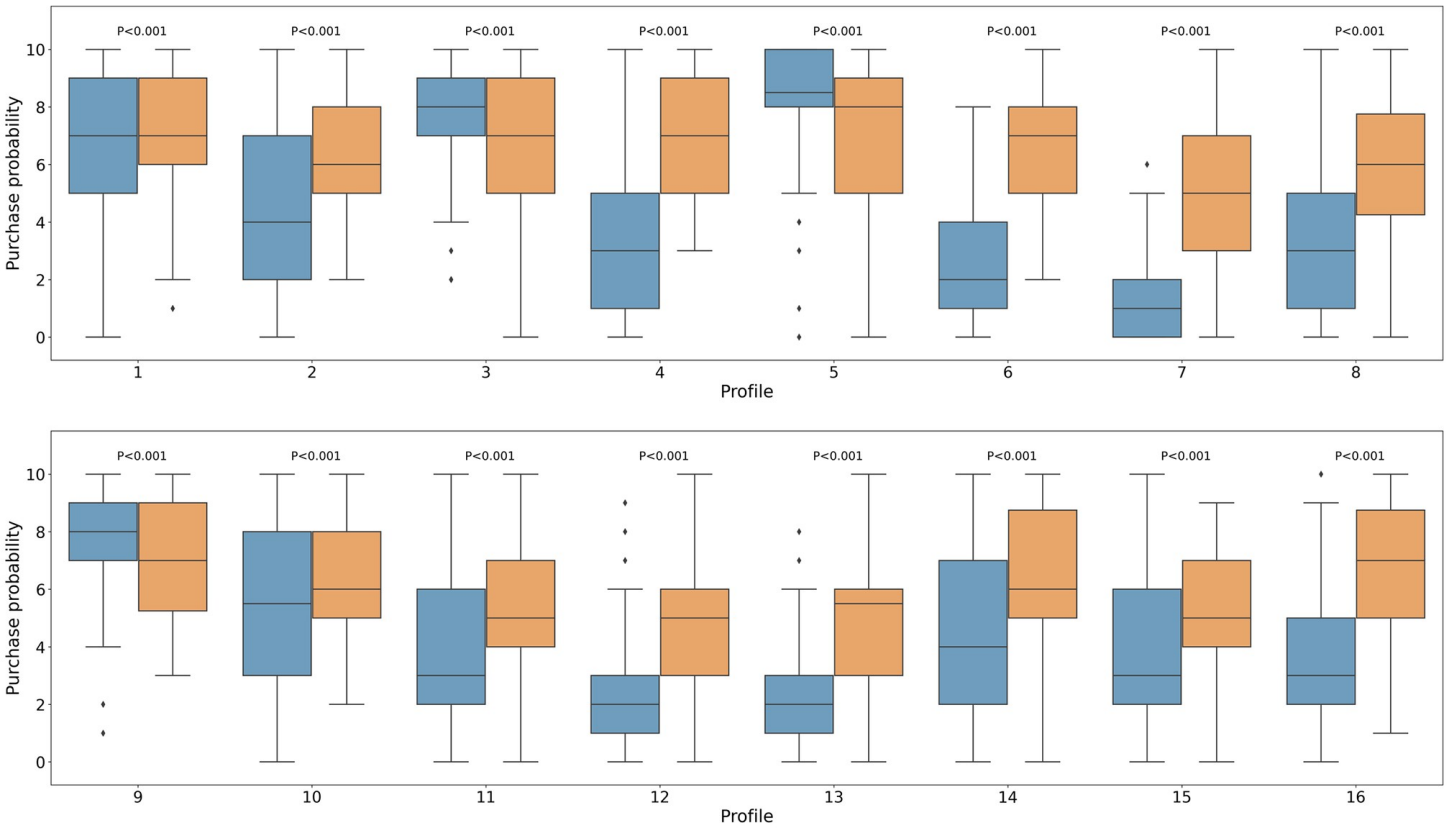
Box plots of purchase probability per profile by cluster. *Notes*: Blue and orange boxes are for cluster 1 and cluster 2, respectively. The numbers between the two boxes denote p-values of the results of t-test to confirm whether the average purchase probabilities between cluster 1 and 2 are different or not.

In addition, we calculated the average and distribution of purchase probability for each profile among the full and subsamples (Table 7, Fig 1; for the attribute levels of each profile, see Table 3).

**Table 7:** Average purchase probability per profile by cluster.

Cluster 1 (n = 76)						Cluster 2 (n = 54)					
Profile	Average	Rank	Profile	Average	Rank	Profile	Average	Rank	Profile	Average	Rank
1	6.71	4	9	7.99	2	1	6.83	5	9	7.06	2
2	4.32	7	10	5.47	5	2	6.31	9	10	6.37	7
3	7.88	3	11	3.88	8	3	6.41	6	11	5.37	12
4	3.66	10	12	2.17	14	4	7.15	1	12	4.52	16
5	8.01	1	13	2.12	15	5	6.93	3	13	5.24	13
6	2.36	13	14	4.33	6	6	6.35	8	14	6.20	10
7	1.43	16	15	3.79	9	7	4.70	15	15	5.04	14
8	3.55	12	16	3.63	11	8	5.96	11	16	6.89	4

In clusters 1 and 2, average purchase probability was the highest for profiles 5 and 9 for cluster 1 (8.01 and 7.99, respectively) and profiles 4 and 9 for cluster 2 (7.15 and 7.06, respectively). The ranks of average purchase probability for the remaining profiles varied across the two clusters: decreasing in the order of profiles 3, 1, 10, 14, and 2 in cluster 1; and in the order of profiles 5, 11, 1, 3, and 10 in cluster 2. Therefore, purchase intention for the same profile (i.e., same attribute levels) differed across clusters, signifying that researcher segments were distinct in willingness to pay for the utility of each attribute level.

We used boxplots (Fig 1) to visualize the distribution of purchase probability per profile for the full sample and subsamples (clusters 1 and 2). Purchase probabilities for a given profile fell between the first (Q1, 25%) and third quartiles (Q3, 75%). The line in the boxes indicate median purchase probabilities for a given profile. The top and bottom lines are the maximum and minimum values of the purchase probabilities for a certain profile. Fig 1 shows the distribution of purchase probability per profile for the subsamples: cluster 1 (76 respondents) and 2 (54 respondents). Boxplots of clusters 1 and 2 are expressed in blue and orange, respectively. Notably, the boxplot of cluster 1 indicates that the width between the maximum value and Q3 for profile 2 is greater than the width between the minimum value and Q1, meaning that observed values are distributed more densely at the bottom. Conversely, the boxplot of profile 2 in cluster 2 shows that the width between the maximum value and Q3 is smaller than the width between the minimum value and Q1, implying that the observed values are mostly distributed at the top. The two boxplots of profile 2 in clusters 1 and 2 show that the width between Q1 and Q3 is small, with the median located slightly below the two; therefore, purchase probability is distributed near the center.

## Discussion

This study used a conjoint analysis to explore the effects of attributes and their respective levels on researcher preferences for chemical compounds provided by the KCB. The part-worth utility for each level of all attributes and important attribute values were estimated from surveying Korean researchers using KCB-provided chemical compounds. We also classified user segmentation through cluster analysis.

Our analysis has important implications. First, from a utilitarian disciplinary perspective, we showed that researchers gain the highest part-worth utility when the structure database of chemical compounds is provided, followed by offering chemical compounds with high new functionality, screening services for chemical compounds, inexpensive chemical compounds, drug action data provided only by the KCB, and drug action data found only in the literature. The biotechnology sector is a high-value-added industry with a large proportion of R&D. The likelihood of technological advancement causes major countries, including the United States, United Kingdom, Japan, and Germany, to implement policies that support and foster the biotechnology sector. However, passive investments are also common owing to the high-risk nature of this sector. Although the economic value of bioindustry is high, the economic value of chemical compounds is vague, raising doubts about their utility relative to investment. Moreover, Korea has a smaller budget than other developed countries. Therefore, considering the results of this study, the part-worth utility estimates of each attribute level, can help the Korean government and KCB efficiently and effectively implement demand-driven policy strategies to promote the utilization of chemical compounds for R&D of new pharmaceutical drugs, thereby enhancing innovation-based performance and promoting commercialization in the pharmaceutical industry.

Second, this study finds convincing evidence that the researchers view “structure database” and “new functionality” as more important in their decision-making about the R&D use of chemical compounds than other attributes. This suggests that it is necessary for the KCB to conduct internal and external organizational R&D activities, such as the exploration of structure, the development and construction of structure databases, and the exploration of new functionalities, to provide chemical compounds suitable for researchers’ needs. Such organizational R&D activities require huge investments in technology and bioinformatics infrastructure. Therefore, it is challenging for the government and KCB to focus on R&D activities to tackle the two attributes, structure database and new functionality simultaneously. This pressures the government and KCB to devise effective and efficient policy and management strategies for R&D when the budget is small.

Third, the cluster analysis results of this study show that users are separated into two groups: cluster 1 shows that the dominant appealing attributes for 76 researchers are structure (attribute importance of 41.2%) and high new functionality (attribute importance of 33.5%); cluster 2 shows that the dominant appealing attributes for 54 researchers are high new functionality (attribute importance of 41.0%) and price (attribute importance of 18.6%). The cluster analysis results suggest that from the economies-of-scale perspective, the KCB should focus on the researchers’ needs in cluster 1. Therefore, management strategies toward promoting R&D activities that induce exploration of compound structures and the development and construction of such information databases should receive the highest priority. This should be followed by R&D activities that promote the exploration of new functionality of chemical compounds. These two types of R&D activities contribute to enhancing the effectiveness and efficiency of KCB management by targeting clusters 1 and 2. However, policymakers and managers should keep in mind that it does not necessarily mean that excluding price in cluster 1 and type in cluster 2, which are statistically insignificant in the estimation of part-worth utility function, the remaining attributes do not promote the use of chemical compounds.

Fourth, this study shows the average purchase probability of each profile card from researchers. Purchase probability implies that researchers are willing to buy a particular chemical compound with a combination of attributes that affect researchers’ preferences. Therefore, the purchase probability of subsamples (clusters 1 and 2) provides information about the order of a chemical compound that KCB attempts to appeal to researchers’ needs. For example, profile cards 5 and 9 for cluster 1 and 4, and 9 for cluster 2, had the highest average purchase probability from researchers: Profile card 5 is a chemical compound with a combination of improved new functionality, deposition by researchers, structure database, drug action data only published in literature, offer of screening services, and a price of 300,000 KRW. Profile card 9 is a chemical compound with a combination of high new functionality, bought abroad, structure database, no drug action data, offer of screening service, and a price of 100,000 KRW. Profile card 4 is a chemical compound with a combination of improved new functionality, deposition by researchers, no structure database, no drug action data, offer of screening services, and a price of 40,000 KRW. The results suggest that KCB develops a product mix (the set of chemical compounds that KCB offers researchers for R&D) based on the combination of attribute levels with high purchase probability, promoting the use of chemical compounds.

## Conclusions

This study proposes a systematic approach to evaluate researcher preferences for KCB-provided chemical compounds. Drawing on survey data, we conducted a conjoint analysis to estimate the part-worth utility for each attribute level and calculate relative importance of attributes. Furthermore, we employed the HB technique to obtain individual-level part-worth estimates and performed estimates-based cluster analysis to group the respondents with similar part-worths or importance values to identify segments. The present study is the first to explore researcher preferences for chemical compounds used in drug development. Unlike most studies that identify factors influencing successful drug development or develop a method for measuring drug development costs, we applied an economics-based, utilitarian approach to evaluate researcher preferences for chemical compounds. Because drug development starts with the selection of chemical compounds, researcher preferences have a strong influence on the compounds used during R&D. We found that part-worth utilities of attribute levels influenced researcher preferences and purchase probability, suggesting that KCB should actively consider these characteristics to maximize the use of chemical compounds. Furthermore, our findings benefit policymakers and managers of public chemical banks, allowing them to better promote the use of chemical compounds and implement demand-induced policy strategies. The resultant enhancement in R&D efficiency should lead to success and improved commercialization for the pharmaceutical industry.

Although this study improves our understanding of the potential mechanisms underlying researcher preferences for chemical compounds in pharmaceutical R&D, it has several limitations. First, this study is specific to Korea and the results may not be generalizable to other countries. Therefore, these relationships should be tested on more chemical compounds in different countries to verify their wider applicability. Second, this study classified researchers into two segments based on individual-level part-worth utilities calculated using the HB method. However, the structure of segment membership depended on researcher behavior, and our study did not consider behavioral variables. Therefore, future research should aim to incorporate researcher-segmentation data. Third, this study did not focus on researcher use of chemical compounds in drug development for specific diseases. Our analysis could be further elaborated by including this angle, analyzing whether utility preferences would change when researchers are focused on compounds targeting different diseases, such as anticancer, anti-inflammatory, and antiviral agents. Fourth, future studies should continue to improve the efficiency of conjoint questions. Here, we formed a focus group of researchers using chemical compounds from KCB to generate the appropriate attributes and levels for conjoint analysis. Our respondents did not ask for improvements to the conjoint questions during the preliminary survey, nor did preliminary results reveal any reversals. Although these outcomes validate the conjoint questions to some extent, we note that our preliminary survey was conducted with only one questionnaire containing the same questions for all respondents. Using multiple questionnaires with different attributes and levels can generate improved attributes and levels for chemical compounds.

## Supporting information

S1 Table Distribution characteristics of users by cluster. (DOCX)
