# Supplementary material for: What drives researcher preferences for chemical compounds? Evidence from conjoint analysis
Source: PLoS One. 2023 Nov 27;18(11):e0294576. doi: 10.1371/journal.pone.0294576 (PMC10681187; doi:10.1371/journal.pone.0294576)
Supplement: S1 Table — (DOCX) [file pone.0294576.s001.docx]

**S1 Table. Distribution characteristics of users by cluster.**

| Division | | Cluster 1 (n = 76) | | Cluster 2 (n = 54) | |
| --- | --- | --- | --- | --- | --- |
|  |  | Frequency | Ratio (%) | Frequency | Ratio (%) |
| Gender | Male | 48 | 63.2 | 43 | 79.6 |
|  | Female | 28 | 36.8 | 11 | 20.4 |
| Age | 20-29 | - | - | 1 | 1.9 |
|  | 30-39 | 15 | 19.7 | 14 | 25.9 |
|  | 40-49 | 27 | 35.5 | 17 | 31.5 |
|  | 50-59 | 25 | 32.9 | 17 | 31.4 |
|  | Over 60 | 9 | 11.9 | 5 | 9.3 |
| Institution type | University | 25 | 32.9 | 17 | 31.5 |
|  | Firm | 17 | 22.4 | 9 | 16.7 |
|  | Research Institute | 34 | 44.7 | 28 | 51.8 |
| Main use of chemical compounds | For R&D | 59 | 77.6 | 44 | 81.5 |
|  | For commercial use | 2 | 2.7 | 1 | 1.8 |
|  | For R&D and commercial use | 15 | 19.7 | 9 | 16.7 |
